# Phenytoin Cream for the Treatment of Neuropathic Pain: Case Series

**DOI:** 10.3390/ph11020053

**Published:** 2018-05-28

**Authors:** David J. Kopsky, Jan M. Keppel Hesselink

**Affiliations:** 1 Institute for Neuropathic Pain, Vespuccistraat 64-III, 1056 SN Amsterdam, The Netherlands; 2 Institute for Neuropathic Pain, Spoorlaan 2a, 3735 MV Bosch en Duin, The Netherlands; jan@neuropathie.nu

**Keywords:** topical, phenytoin, neuropathic pain, treatment, cream

## Abstract

BACKGROUND: Neuropathic pain can be disabling, and is often difficult to treat. Within a year, over half of all patients stop taking their prescribed neuropathic pain medication, which is most probably due to side effects or disappointing analgesic results. Therefore, new therapies are needed to alleviate neuropathic pain. As such, topical analgesics could be a new inroad in the treatment of neuropathic pain. In 2014, we developed a new topical formulation containing either phenytoin or sodium phenytoin. After optimization of the formulation, we were able to reach a 10% concentration and combine phenytoin with other co-analgesics in the same base cream. OBJECTIVE: To describe a series of 70 neuropathic pain patients who were treated with phenytoin cream. MATERIAL AND METHODS: Cases treated with phenytoin 5% or 10% creams were gathered. The mean onset of pain relief, the duration of effect, and reduction in pain intensity measured on the 11-point numerical rating scale (NRS) were all studied. A single-blind response test with phenytoin 10% and placebo creams was conducted on 12 patients in order to select responders prior to prescribing the active cream. Plasma phenytoin concentrations were measured in 16 patients. RESULTS: Nine patients applied phenytoin 5% cream, and 61 patients used phenytoin 10% cream. After grouping the effects of all of the patients, the mean onset of pain relief was 16.3 min (SD: 14.8), the mean duration of analgesia was 8.1 h (SD: 9.1), and the mean pain reduction on the NRS was 61.2% (SD: 25.0). The mean pain reduction on the NRS while using phenytoin cream was statistically significant compared with the baseline, with a reduction of 4.5 (CI: 4.0 to 5.0, *p* < 0.01). The 12 patients on whom a single-blind response test was performed experienced a statistically significant reduction in pain in the area where the phenytoin 10% cream was applied in comparison to the area where the placebo cream was applied (*p* < 0.01). Thirty minutes after the test application, the mean pain reduction on the NRS in the areas where the phenytoin 10% cream and the placebo cream were applied was 3.3 (CI: 2.3 to 4.4, *p* < 0.01) and 1.1 (CI: 0.4 to 1.9, *p* < 0.05), respectively. In all 16 patients, the phenytoin plasma levels were below the limit of detection. So far, no systemic side effects were reported. Two patients only reported local side effects: a transient burning aggravation and skin rash. CONCLUSION: In this case series, the phenytoin cream had reduced neuropathic pain considerably, with a fast onset of analgesic effect.

## 1. Introduction

Neuropathic pain can be disabling, and is often difficult to treat [1,2]. Guidelines for neuropathic pain treatment only provide for a limited number of therapies, and the numbers needed to treat most interventions are disappointing—between six and 10—indicating that many patients remain in pain even after being prescribed various analgesics [3]. More than half of the patients stop using neuropathic pain medication within a year, most probably because of side effects and/or disappointing clinical results [4]. Topical analgesics are therefore an interesting therapeutic option to reduce neuropathic pain with a low risk of systemic side effects [5]. However, up to now, only lidocaine 5% plasters and capsaicin 8% plasters are registered for the treatment of neuropathic pain. Capsaicin 8% plasters have to be applied in an outpatient clinic every three months, and lidocaine 5% plasters can be troublesome in handling, especially when applied on the feet of elderly patients. Therefore, since 2009, we decided to develop various compounded creams containing one or more active pharmaceutical ingredients (APIs) [6]. We subsequently reported the effects of amitriptyline, ketamine, baclofen and related co-analgesics in such creams [7,8,9,10,11,12,13].

Due to the fast onset of pain relief after the application of creams containing such co-analgesics and the symmetrical distribution of pain intensity in case of a peripheral neuropathy (e.g., comparable pain scores in left and right foot), we were able to develop a test to identify those who responded to the cream within 30 min after application. During the first phase of data collection, we performed an open response test, applying the active cream on one foot. The patient was labeled a responder after a pain reduction of two points or more on the 11-point numerical rating scale (NRS) within a period of 30 min in the area where the active cream was applied. To control for the placebo effect, we developed a single-blind response test comparing an active cream with a placebo cream; the test takes only a minute to conduct. The single-blind response test could be performed when there was maximally one-point difference on the NRS between two painful areas (e.g., both feet) at baseline. Patients would then administer an equal amount of placebo cream and active cream on each foot using different hands to avoid contamination. Responders were defined as patients reporting (1) within 30 min; (2) ≥two-point pain reduction on the NRS in the active cream applied area; and (3) ≥one-point difference on the NRS between the active cream and the placebo cream area. A two-point reduction on the NRS was seen as clinically relevant [14].

**Figure 1:**
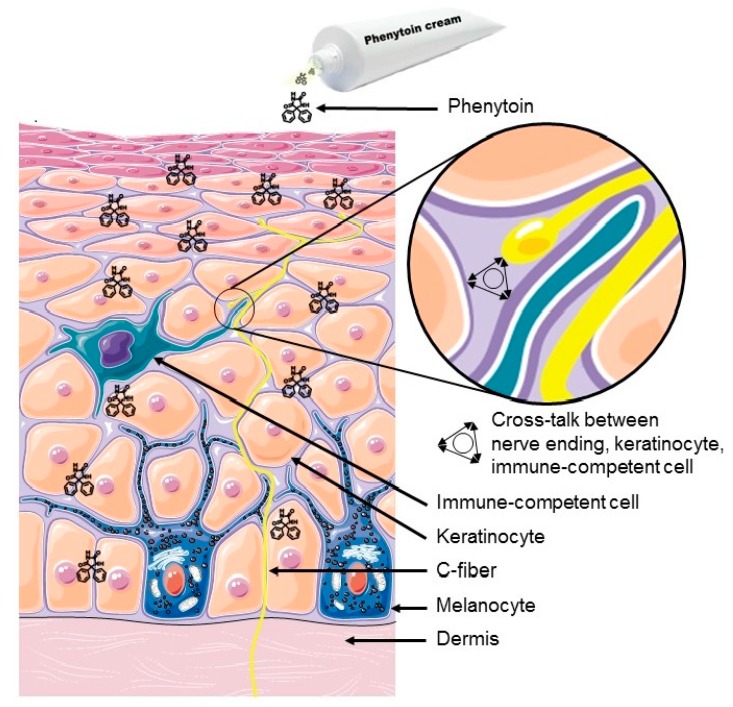
Cross-talk between nerve ending, keratinocyte, and an immune-competent cell.

Although many patients were responders to compounded creams containing for example amitriptyline, ketamine, and baclofen, a number of patients remained treatment-resistant. We therefore introduced phenytoin as a new compound for topical neuropathic pain treatment based on its remarkable history and its various modes of action [15,16,17]. Phenytoin, as a broad-acting ion channel blocker with neuroprotective and anti-inflammatory properties, seemed to be an optimal choice as an effective compound that is able to modulate peripheral mechanisms, which are increasingly recognized as important drivers in neuropathic pain [18]. In diabetic neuropathy, chronic idiopathic axonal polyneuropathy (CIAP), small fiber neuropathy (SFN), and chemotherapy induced peripheral neuropathy (CIPN), pain generators appear to reside in the skin, especially in the epidermis. These targets contribute to peripheral sensitization, and may result from the cross-talk between three different players: the nerve endings of nociceptors, the keratinocytes, and the immune-competent cells (Figure 1) [19,20]. All of these components are known to express sodium channels of different classes (NaV1.3-1.5,1.7,1.8) [21]. Thus, a broad-acting sodium-channel blocking agent would be most suited for use as the API in a compounded cream for the topical treatment of neuropathic pain. Since we first introduced the cream in our clinic in 2014, we began gathering detailed clinical information on newly-treated patients on a regular basis. We now present this cohort, which consists of 70 patients who are being treated with a phenytoin 5% and/or 10% cream.

## 2. Material and Methods

Data from those patients using phenytoin 5% or 10% creams were gathered from January 2014 to October 2017, and the pain intensity was measured on the NRS. Descriptive statistics were used for socio-demographic data, diagnoses, and pain characteristics. The screenings tool for neuropathic pain (DN-4: Douleur Neuropathique, four questions) was used to determine the pain characteristics [22]. We calculated the mean, standard deviation, and range for pain intensity before a phenytoin cream application; we also measured the onset of pain relief, the duration of effect, the duration of treatment, and the percentage of pain reduction on the NRS. The non-parametric Wilcoxon signed-rank tests were performed for pre-test/post-test comparisons concerning pain reduction on the NRS from baseline. In the single-blind response test group, these tests were performed comparing the mean pain reduction between the phenytoin 10% and placebo cream areas. We calculated the number of patients achieving minimum pain relief (MPR) from the baseline of 30% (moderate pain relief: MPR30), 50% (considerable pain relief: MPRC50), and 70% (robust pain relief: MPR70) measured on the NRS [23]. We used independent t-tests to analyze the differences between two different groups (phenytoin 10% and phenytoin 5% users). The statistical analysis was performed with SPSS 22 (SPSS Inc., Chicago, IL, USA). We collected plasma for the determination of phenytoin levels in a sub-cohort of 16 patients. Blood was taken around the estimated T-max, between 1–3 h after the application of the cream.

## 3. Results

From January 2014 to October 2017, patients were treated with phenytoin 5% or 10% cream. Outcome from the 70 patients was documented in detail. The mean duration of neuropathic pain was 7.8 years (SD: 6.5). Thirty-four patients were male (48.6%), and 36 (51.4%) were female. The age of the patients ranged between 43–89 years, with a mean age of 67.7 years (SD: 10.4). The diagnoses of those patients treated with phenytoin 5% or 10% cream are summarized in Table 1. Nine patients used the phenytoin 5% cream, and 61 patients used the 10% cream. The mean NRS before the use of phenytoin cream for the whole group was 7.4 (SD: 1.1). The mean pain reduction on the NRS while using phenytoin cream was statistically significant, with a reduction of 4.5 (CI: 4.0 to 5.0, *p* < 0.01).

An open or single-blind response test was performed on 10 or 12 patients, respectively. To date, the patients described in this cohort have been, and continue to be, treated over a period of a few weeks to 41 months, and most did not experience any side effects. Four patients have stopped using phenytoin cream, because their pain has been resolved.

Patients experiencing certain neuropathic pain characteristics as defined by the DN-4 are presented in Table 2. Fifty patients (71%) experienced three or more neuropathic pain characteristics. The 12 patients on whom a single-blind response test was performed scored their baseline pain intensity as 6.6 on the NRS (SD: 1.1) in the area where the phenytoin 10% cream had been applied, and 6.5 (SD: 1.2) in the area where the placebo cream had been applied. Thirty minutes after the test application, the pain reduction on the NRS in both the phenytoin 10% cream-applied area and placebo-applied area was 3.3 (CI: 2.3 to 4.4, *p* < 0.01) and 1.1 (CI: 0.4 to 1.9, *p* < 0.05), respectively. This difference was statistically significant (*p* < 0.01). While using phenytoin 10% cream during the open treatment phase, patients experienced a comparable pain reduction of 4.0 points on the NRS (CI: 2.8 to 5.1, *p* < 0.01), with 7.3 (SD: 0.4) as baseline.

Table 3 summarizes the various parameters that are related to the application of phenytoin cream. Table 4 shows more detailed information of those patients using phenytoin 10% cream with the four most common diagnoses: Painful Diabetic Neuropathy, CIAP, PN of unknown origin, and SFN. Interestingly, the mean duration of effect after the phenytoin 10% cream application differs significantly between SFN (10.7 h, SD: 5.1) and CIAP (6.0 h, SD: 3.1). The difference is almost 5 h (*t*(16) = 2.4, *p* < 0.03), using the independent t-test. The onset of action after the phenytoin 10% cream application in CIAP patients is almost 14 min longer than in SFN patients. However, this difference does not reach significance using the independent *t*-test (*t*(15) = 1.7, *p* = 0.1). More than one third of the patients experienced a robust relief of pain (MPR70).

The mean onset of action (onset to perceptible pain relief) after application was about 15 min for both phenytoin 5% and 10% creams. The mean duration of analgesia was almost 5 h for the phenytoin 5%, while patients treated with the phenytoin 10% cream reported a mean duration of meaningful analgesia of more than 8 h. However, the difference of 3.8 h between the two groups was not statistically significant (*t*(66) = 1.2, *p* = 0.2) when using the independent *t*-test.

Only two patients reported local side effects: transient aggravation of a burning sensation, and one patient experienced red papules after the administration of the phenytoin 10% cream, though in that case not after the application of the phenytoin 5% cream. These side effects were transient, and disappeared after treatment stopped. Plasma samples were taken after several days of the application of the phenytoin 10% cream in 16 patients. 

## 4. Plasma Sampling

Up until now, phenytoin plasma levels were measured after the application of the phenytoin 10% cream in 16 patients. As patients traveled to our Institute for Neuropathic Pain from all parts of the Netherlands, we gave instructions to measure phenytoin plasma levels after the phenytoin application at their local clinical laboratories. The standard phenytoin plasma determination was performed with high-performance liquid chromatography, and determined the plasma protein-bound phenytoin concentration, which was approximately 90% of the total phenytoin in plasma [24]. Most of the patients applied the phenytoin cream for a period of one to two weeks before the measurement, and the sampling time was usually 1.5 h to 3 h after the last application. One patient performed a blood sampling 104 days after continuously using the phenytoin 10% cream. The mean daily application was 2.3 grams (SD: 1.7). No phenytoin plasma levels were detected (all of the measures were below the limit of detection), even after the application of 6.7 grams of the phenytoin 10% cream in one case.

## 5. Discussion

This case collection indicates that phenytoin cream can be helpful in the treatment of neuropathic pain. The fast onset of action of the phenytoin cream enables us to differentiate responders from non-responders via a single-blind response test based on both placebo and active creams, which were applied on two different localized pain areas. This method has the advantage that we can prescribe an active analgesic cream only to responders, and thus individualize therapy directly. Furthermore, such a targeted prescription will reduce the chances that patients would end up as non-responders to the cream, after an initial placebo response of some weeks. For most oral neuropathic pain medication, the onset of action takes days to weeks, which patients report as being a much longer period than when treated with phenytoin cream. Both capsaicin 8% plasters and oral pregabalin have a median onset of action (≥30% pain reduction) of 7.5 days and 36 days, respectively [25]. Another study showed that pregabalin delivered a significant pain reduction at day two [26]. Also at day two, gastro-retentive gabapentin showed a significant pain relief over placebo [27]. For the lidocaine 5% patch, the onset of action was reported within 4 h [28]. The onset of action of an average of 15 min is therefore very fast. This might be due to the effects of phenytoin on the nerve endings in the epidermis, and also the possible effect on the keratinocytes and the immune competent cells, all of which cross-talk with the nerve endings of nociceptors [19]. Especially in the group of SFN patients, the onset of action is very fast: less than 10 min. This might be due to the contribution of the epidermal pathology in this disease. We found a difference between the action of onset and the duration of effect between CIAP patients and SFN patients; this might suggest a difference in pathogenetic pathways, which are perhaps related to a more dominant intradermal pathology in SFN patients compared with CIAP patients. As CIAP patients most probably belong to different pathogenetic subgroups, our data also suggest there might be two cohorts of CIAP patients: one based on dominant intradermal small fiber nerve pathology, and those less dependent on such pathology.

The percentage of patients experiencing a pain reduction of at least 50% and 70% on the NRS is around 65% and 35%, respectively. This high percentage of responders is most probably due to the open nature of this case series, and might also be related to the installment of the response test in 20 patients. However, topical analgesics are known to induce a high placebo response [29]. That we could achieve a statistically significant difference between the phenytoin 10% cream effects in comparison to the placebo cream in the single-blind response test phase further supports the use of the cream. 

The average duration of the analgesic effect of phenytoin 10% cream is over 8 h, which corresponds with a mean daily application of 2.4 times. Patients can therefore apply phenytoin cream in the morning and evening, and if needed also at noon, to cover the whole day. The lack of a statistically significant difference in the duration of analgesia between the phenytoin 5% and 10% creams might be due to the small sample size.

No systemic side effects have been reported. This finding is in line with the phenytoin plasma levels being below the limit of detection in 16 patients in our data pool. Previous studies did not show either detectable phenytoin plasma concentrations after applying the 2% and 5% creams on skin lesions due to epidermolysis bullosa simplex or sprinkling up to 400 mg of phenytoin sodium salt (100%) daily into wounds [30,31]. 

Only two patients reported local side effects: a transient aggravation of burning sensation, and in one patient, red papules after the administration of the phenytoin 10% cream, although in that case, not after the application of the phenytoin 5% cream. These local side effects were transient, and disappeared after stopping treatment.

## 6. Conclusions

Phenytoin cream seems safe and effective in a cohort of 70 patients. As most patients were non-responders or partial responders to the oral analgesic interventions described in the guidelines, our data suggest that such a cream might become a new tool in our armamentarium of therapies for peripheral neuropathic pain. In most patients, the onset of analgesic action is fast, around 15 min. This is of particular interest, as most oral analgesics can take days to weeks before sufficient pain reduction sets in. Furthermore, the fast onset of action makes a single-blind placebo controlled response test possible. Thus, this test enables the clinician to identify responders directly at the first visit, and subsequently to prescribe the cream. Such an approach, together with the fast onset of action, might also enhance compliance. Side effects were rare. The open nature of this data collection limits the ability to make firm conclusions, and in the future, well-controlled studies are required to analyze the efficacy and safety of this topical phenytoin formulation in more detail. Furthermore, the statistically significant effects of the phenytoin 10% versus the placebo cream in the single-blind response test population supports further explorations. New studies are currently in the design phase, both in CIAP patients, as well as in patients suffering from painful diabetic neuropathy.

## Figures and Tables

**Table 1:** Neuropathy diagnoses and summary of treatment regimens.

Diagnosis	Phenytoin 5% N (%)	Phenytoin 10% N (%)
Painful Diabetic Neuropathy	2 (22.2)	8 (13.1)
Chronic Idiopathic Axonal Polyneuropathy	4 (44.4)	11 (18.0)
Peripheral neuropathy of unknown origin	1 (11.1)	9 (14.8)
Small Fiber Neuropathy		8 (13.1)
Chemotherapy-Induced Polyneuropathy		6 (9.8)
Post-Herpetic Neuralgia		6 (9.8)
Peripheral neuropathy due to Lyme disease		1 (1.6)
Trigeminal neuralgia		1 (1.6)
Incomplete spinal cord injury neuropathy	1 (11.1)	1 (1.6)
Meralgia Paresthetica		1 (1.6)
Post toxic axonal polyneuropathy		1 (1.6)
Traumatic neuropathy		1 (1.6)
HMSN type II		1 (1.6)
Polyneuropathy due to vitamin B6 intoxication		2 (3.3)
Radiculopathy		1 (1.6)
Compression neuropathy		1 (1.6)
Plexopathy		1 (1.6)
Complex Regional Pain Syndrome type II	1 (11.1)	1 (1.6)

HMSN: hereditary motor and sensory neuropathy.

**Table 2:** Pain characteristics of neuropathic pain.

Pain Characteristics	Phenytoin 5% N (%)	Phenytoin 10% N (%)
Burning	8 (88.9)	51 (83.6)
Painful cold	3 (33.3)	12 (19.7)
Electric shocks	2 (22.2)	17 (27.9)
Tingling	8 (88.9)	39 (63.9)
Pins and needles	7 (77.7)	43 (70.5)
Itch	1 (11.1)	10 (16.4)
Allodynia	3 (33.3)	25 (41.0)
Cramps	1 (11.1)	6 (9.8)

**Table 3:** Use and effect characteristics of phenytoin cream.

Use Characteristics and Effect	Phenytoin 5% & 10% Mean (SD), [Range]	Phenytoin 5% Mean (SD), [Range]	Phenytoin 10% Mean (SD), [Range]
Number of daily application	2.4 (1.3) [0.1–7.0]	2.7 (1.3) [1.0–5.5]	2.4 (1.3) [0.1–7.0]
Grams per application	0.9 (0.9) [0.2–6.7]	0.8 (0.3) [0.6–1.2]	0.9 (0.9) [0.2–6.7]
Grams of daily application	1.9 (1.3) [0.1–6.7]	2.0 (0.7) [0.9–3.3]	1.9 (1.4) [0.1–6.7]
Onset of action (minutes)	16.3 (14.8) [1–60]	15.2 (17.7) [1–60]	16.5 (14.4) [2–60]
Duration of effect (hours)	8.1 (9.1) [1–70]	4.8 (2.2) [2.5–10]	8.6 (9.7) [1–70]
Duration of use (months)	3.6 (5.3) [0.5–41]	3.0 (2.4) [1–9]	3.6 (5.6) [0.5–41]
NRS reduction in %	61.2 (25.0) [12.5–100]	65.8 (26.4) [35.7–100]	60.6 (24.9) [12.5–100]
MPR30	64 (91.4%)	9 (100%)	55 (90.2%)
MPR50	49 (70.0%)	6 (66.7%)	43 (70.5%)
MPR70	25 (35.7%)	4 (44.4%)	21 (34.4%)

MPR: number of patients reaching a minimum pain relief of 30%, 50% and 70% from baseline; NRS: 11-point numerical rating scale, SD: standard deviation.

**Table 4:** Characteristics and effect of phenytoin 10% cream in the four most seen indications.

	DM II (N = 8)	CIAP (N = 11)	PN u.o. (N = 9)	SFN (N = 8)
Duration NP, years (SD)	10.0 (6.2)	9.4 (4.8)	6.1 (3.1)	5.3 (5.1)
Male/Female, N	6/2	5/6	4/5	4/4
Age, mean (SD)	69.9 (8.9)	67.5 (6.5)	72.8 (11.6)	65.3 (10.4)
Burning, N (%)	6 (75%)	8 (72.7%)	8 (88.9%)	8 (100%)
Painful cold, N (%)	2 (25%)	2 (18.2%)	2 (22.2%)	1 (12.5%)
Electric shocks, N (%)	3 (37.5%)	3 (27.3%)	2 (22.2%)	8 (100%)
Tingling, N (%)	7 (87.5%)	8 (72.7%)	5 (55.6%)	2 (25%)
Pins and needles, N (%)	8 (100%)	9 (81.8%)	5 (55.6%)	3 (37.5%)
Itch, N (%)	0	1 (9.1%)	0	1 (12.5%)
Allodynia, N (%)	4 (50%)	6 (54.5%)	4 (44.4%)	8 (100%)
Cramps, N (%)	0	3 (27.3%)	0	1 (12.5%)
Number of daily applications, mean (SD)	2.1 (0.5)	2.1 (1.0)	1.8 (0.7)	1.7 (0.6)
Grams per application, mean (SD)	1.0 (0.4)	1.0 (0.4)	1.0 (0.6)	1.5 (2.1)
Grams of daily application, mean (SD)	2.1 (1.0)	2.1 (1.5)	1.7 (1.2)	2.0 (2.1)
Onset of action in minutes, mean (SD)	11.7 (9.3)	23.5 (21.3)	17.4 (18.2)	9.7 (4.3)
Duration of effect in hours, mean (SD)	7.0 (3.9)	6.0 (3.1)	10.4 (7.1)	10.7 (5.1)
Duration of use in months, mean (SD)	6.3 (13.1)	2.9 (2.8)	3.4 (1.8)	2.1 (0.9)
	N (%)	N (%)	N (%)	N (%)
MPR30	8 (100%)	9 (81.8%)	9 (100%)	8 (100%)
MPR50	8 (100%)	7 (63.6%)	5 (55.6%)	7 (87.5%)
MPR70	3 (37.5%)	2 (18.2%)	3 (33.3%)	2 (25%)

NP: neuropathic pain; DM II: diabetes mellitus type II; CIAP: chronic idiopathic axonal polyneuropathy; PN u.o.: peripheral neuropathy of unknown origin; SFN: small fiber neuropathy; SD: standard deviation; MPR: number of patients reaching a minimum pain relief of 30%, 50%, and 70% from baseline.
